# Grants Recommended by the Convalescent Homes Committee

**Published:** 1916-12-23

**Authors:** 


### Grants Recommended by the Convalescent Homes Committee.

### A.—CONSUMPTIVE SANATORIA.

*The Convalescent Homes Committee desire to draw attention to the fact that it must not be assumed that the reduction or absence of a grant implies dissatisfaction. (See paragraph 3 of Report.)*

CHILDREN'S Sanatorium, Holt, Norfolk, £585. In consideration of the reservation of nine children's beds during 1917 for the use of certain London hospitals (see paragraphs 5 and 6 of Report).

DANESWOOD Sanatorium (Jewish), Woburn Sands, £350. In consideration of the reservation of four beds during 1917 for the use of certain London hospitals (see paragraphs 5 and 6 of Report). Devon and Cornwall Sanatorium, Didworthy, South Brent, £875. In consideration of the reservation of ten beds during 1917 for the use of certain London hospitals (see paragraphs 5 and 6 of Report).

EVERSFIELD Chest Hospital, St. Leonards, £50, towards erection of cliff wall.

FAIRLIGHT Sanatorium, Hastings.

KELLING Sanatorium, Holt, Norfolk, £525. In consideration of the reservation of six beds during 1917 for the use of certain hospitals (see paragraphs 5 and 6 of Report).

MOUNT VERNON Hospital, Northwood, £50.

NATIONAL Association for the Establishment and Maintenance of Sanatoria for Workers, Benenden, Kent, £2,050, of which £1,000 in reduction of mortgage debt, and £1,050 in consideration of the reservation of twelve beds during 1917 for the use of certain London hospitals. (see paragraphs 5 and 6 of Report). Northamptonshire Sanatorium, Creaton, Northants, £1,050. In consideration of the reservation of twelve beds during 1917 for the use of certain London hospitals (see paragraphs 5 and 6 of Report).

**Total, £5,535.**

### B.—CONVALESCENT HOMES.

*The names of Convalescent Homes receiving no grant are not published.*

BALDWIN BROWN Home for Convalescent Poor, Herne Bay, £25. Beau Site Convalescent Home, Hastings, £25. Brentwood Convalescent Home for London Children, Brentwood, £50.

CHELSEA Hospital for Women, St. Leonards, £75; with a view to encouraging the admission without payment of patients direct from the hospital. Children's Cottage Hospital, Cold Ash, Newbury, £25; in consideration of convalescent cases. Children's Home Hospital, Barnet, £25; in consideration of convalescent cases. Convalescent Home for Poor Children, St. Leonards, £75. Convalescent Police Seaside Home, Hove, £50.

DEPTFORD Medical Mission Convalescent Home, Bexhill, £25.

FRIENDLY Societies' Convalescent Homes, Dover and Herne Bay, £75.

HIGHGATE Convalescent Home for Children, Highgate, £25.

JEWISH Convalescent Home (Children), Brighton, £50.

KING'S College Hospital, Hemel Hempstead, £75.

METROPOLITAN Convalescent Institution (Children), Broadstairs, £100. Metropolitan Convalescent Institution, Walton, £150.

NATIONAL Hospital for the Paralysed and Epileptic, East Finchley, £100; with a view to facilitating the admission of patients without payment. New Hospital for Women (Home of Recovery), Barnet, £100.

PADDINGTON GREEN Children's Hospital, Slough, £125. Poplar Hospital, Walton-on-Naze, £50.

QUEEN CHARLOTTE'S Lying-in Hospital, Kilburn, £50. Queen's Hospital for Children, Bexhill, £185, of which £35 to reduce debt.

ROYAL Waterloo Hospital for Children and Women, Whippingham, £25.

ST. ANDREW'S Convalescent Home, Folkestone, £75. St. Andrew's Convalescent Hospital, Clewer, £25. St. George's Hospital, Wimbledon, £25. St. Luke's Home (Children), Woodley, Reading, £25; in consideration of convalescent cases. St. Mary's Convalescent Home, Birchington, £25. St. Mary's Home for Children, Broadstairs, £50; in consideration of convalescent cases. St. Michael's Home for Men and Women, Westgate-on-Sea, £25. Sunday School Union Convalescent Home, Bournemouth, £25. Surgical Home for Boys, Banstead, £25; in consideration of convalescent cases.

TILFORD Convalescent Home for Children, Farnham, £15.

VICTORIA Home for Invalid Children, Margate, £25; in consideration of convalescent cases. Victoria Hospital for Children, Chelsea, Broadstairs, £100. Victoria Hospital for Children, Chelsea, Biggin Hill, Kent, £25.

WINIFRED House Invalid Children's Convalescent Hospital Home, Tollington Park, £15 Total, £1,965.

| | |
|---|---|
| **Grants to Consumption Sanatoria ... ...** | **£5,535** |
| **Grants to Convalescent Homes ... ...** | **1,965** |
| **Total Grants by Convalescent Homes Committee for the year 1916 ...** | **£7,500** |

